# Predicting the consequences of global warming on *Gentiana lutea* germination at the edge of its distributional and ecological range

**DOI:** 10.7717/peerj.8894

**Published:** 2020-05-06

**Authors:** Alba Cuena-Lombraña, Marco Porceddu, Caterina Angela Dettori, Gianluigi Bacchetta

**Affiliations:** 1 Sardinian Germplasm Bank (BG-SAR), Hortus Botanicus Karalitanus (HBK), University of Cagliari, Cagliari, Italy; 2 Department of Life and Environmental Sciences, Centre for the Conservation of Biodiversity (CCB), University of Cagliari, Cagliari, Italy

**Keywords:** Base temperatures, Cold stratification, Dormancy, Future climatic scenarios, Gentianaceae, Thermal time

## Abstract

**Background:**

Temperature is the main environmental factor controlling seed germination; it determines both the percentage and the rate of germination. According to the Intergovernmental Panel on Climate Change, the global mean surface temperature could increase of approximately 2–4 °C by 2090–2099. As a consequence of global warming, the period of snow cover is decreasing on several mountain areas. Thermal time approach can be used to characterise the seed germination of plants and to evaluate the germination behaviour under the climate change scenarios. In this study, the effect of different cold stratification periods on seed dormancy release and germination of *Gentiana lutea* subsp. *lutea*, a taxon listed in Annex V of the Habitats Directive (92/43/EEC), was evaluated. Furthermore, the thermal requirements and the consequences of the temperature rise for seed germination of this species were estimated. In addition, a conceptual representation of the thermal time approach is presented.

**Methods:**

Seeds of *G. lutea* subsp. *lutea* were harvested from at least 50 randomly selected plants in two representative localities of the Gennargentu massif (Sardinia). Germination tests were carried out under laboratory conditions and the responses at 5, 10, 15, 20, 25 and 30 °C were recorded. Different cold stratification pre-treatments at 1 ± 1 °C (i.e. 0, 15, 30, 60 and 90 days) were applied. Successively, the base temperature (*T*_b_) and the number of thermal units (θ, °Cd) for germination were estimated. Additionally, this study examined the consequences of an increase in temperatures based on the Representative Concentration Pathways (RPC) scenarios.

**Results:**

The results indicated that from 0 to 30 days of cold stratification, the germination was null or very low. After 60 and 90 days of cold stratification the seed dormancy was removed; however, 25 and 30 °C negatively affected the germination capacity of non-dormant seeds. Seeds cold-stratified for 90 days showed a lower *T*_b_ than those stratified for 60 days. However, 60 and 90 days of cold stratification did not cause great variations in the thermal time units. Analysing the RPC scenarios, we detected that the number of days useful for dormancy release of seeds of *G. lutea* may be less than 30 days, a condition that does not permit an effective dormancy release.

**Conclusions:**

We conclude that seeds of *G. lutea* need at least 60 days of cold stratification to remove dormancy and promote the germination. The thermal time model developed in this work allowed us to identify the thermal threshold requirements of seed germination of this species, increasing the knowledge of a plant threatened by global warming. Our results emphasise the need for further studies aiming at a better characterisation of germination efficiency, especially for species that require cold stratification. This would improve the knowledge on the germination mechanisms of adaptation to different future global warming conditions.

## Introduction

Temperature is the main environmental factor controlling seed germination in moist soils and it determines both the percentage and the rate (understood as velocity) of seeds that germinate (Heydecker, 1977; Baskin & Baskin, 2014). This factor, together with solar radiation and humidity, is one of the main drivers in regulating distribution and growth of mountainous plant species (Körner, 2003). In particular, low soil temperatures play a crucial role for productivity and growth both in alpine and Mediterranean mountain plants (Kramer, Leinonen & Loustau, 2000; Körner, 2003; Körner & Hiltbrunner, 2018). It is also widely accepted that a dominant control on the natural distribution of species is exerted by climate (Pearson & Dawson, 2003). The recent climatic changes have an important influence on the distribution of the species (Pearson & Dawson, 2003) and are affecting a wide range of organisms with different geographical distributions (Hughes, 2000; Wuethrich, 2000; McCarty, 2001). Direct effects of rising temperatures and changes on the reproductive and physiological processes in plants species have been documented over the last decades (Hedhly, Hormaza & Herrero, 2009 and references therein), and recent data (including past and present climatic inputs) reinforce the idea that the reproductive phases (e.g. plant reproduction, the reproductive output of plants, seed germination success and seedling development) are particularly vulnerable to climate change (Fernández-Pascual, Mattana & Pritchard, 2019 and references therein). According to the Intergovernmental Panel on Climate Change (IPCC Team, Pachauri & Meyer, 2014), the global mean surface temperature could increase of approx. 2–4 °C by 2090–2099. In this contest, the IPCC Team, Pachauri & Meyer (2014), evaluating the different estimated greenhouse gas emissions, proposed various Representative Concentration Pathways (RPC) emission scenarios. A stringent mitigation scenario (RCP2.6), two intermediate scenarios (RCP4.5 and RCP6.0) and one scenario with very high greenhouse gas emissions (RCP8.5), with a temperature rise from 0.3 to 4.8 °C, were included in these RCPs (IPCC Team, Pachauri & Meyer, 2014). It is consequently expected that the predicted future climate change will have a remarkable impact on species distribution (IPCC Team, Pachauri & Meyer, 2014). In particular, it has been reported and predicted that a large increase in temperatures will affect the Mediterranean mountain ranges (Peñuelas & Boada, 2003; Nogués-Bravo et al., 2008). In this regard, climate change scenarios (Moss et al., 2010; IPCC Team, Pachauri & Meyer, 2014) can then be used to develop models to predict the changes in the bioclimatic conditions and consequent alterations in composition of plant communities (Berry et al., 2002; Storkey et al., 2014), as well as to quantify germination, dormancy loss and field emergence of wild plant species (Porceddu et al., 2013; Seal et al., 2017).

As a consequence of global warming, defined here as the gradual increase, observed or projected, in global surface temperature (IPCC Team, Pachauri & Meyer, 2014), the period of snow cover is decreasing on several mountain areas around the world (Beniston, Diaz & Bradley, 1997; Beniston, 2012), and further decreases are also expected to occur in the future (García-Fernández et al., 2015). Species with lower temperature threshold requirements for dormancy breaking would be susceptible to increasing temperatures because seeds would remain dormant in the soil seed banks without the requirements of intensity and duration of cold (Ooi, Auld & Denham, 2012). If the current duration of the cold period approaches the minimum requirement, they will not satisfactorily overcome dormancy (Walck et al., 2011). In addition, if temperature continues to warm as a consequence of global warming, our awareness of derived plant reproductive responses will become progressively more significant in the context of future conservation practices for species with seed dormancy (Walck et al., 2011). Therefore, the requirement of a period of cold stratification would allow seeds to sense the presence of snow, resulting in a shift in the germination time to a period which is more appropriate for seedling survival and establishment (Cavieres & Arroyo, 2000; Cavieres & Sierra-Almeida, 2018). Studies carried out on species growing in habitats with unstable snow cover duration and on species with wide altitudinal distribution have shown a positive relationship between the duration of cold stratification (and the snow cover period) and the maximum seed germination (Meyer, McArthur & Jorgensen, 1989; Meyer, Kitchen & Carlson, 1995; Cavieres & Arroyo, 2000; García-Fernández et al., 2015). The need for cold stratification would prevent germination during the unfavourable period (Meyer & Monsen, 1991) and allow germination to occur only after the snowmelt, thus avoiding the adverse effect that freezing temperatures would have on seedlings (Billings & Mooney, 1968).

**Figure 1:**
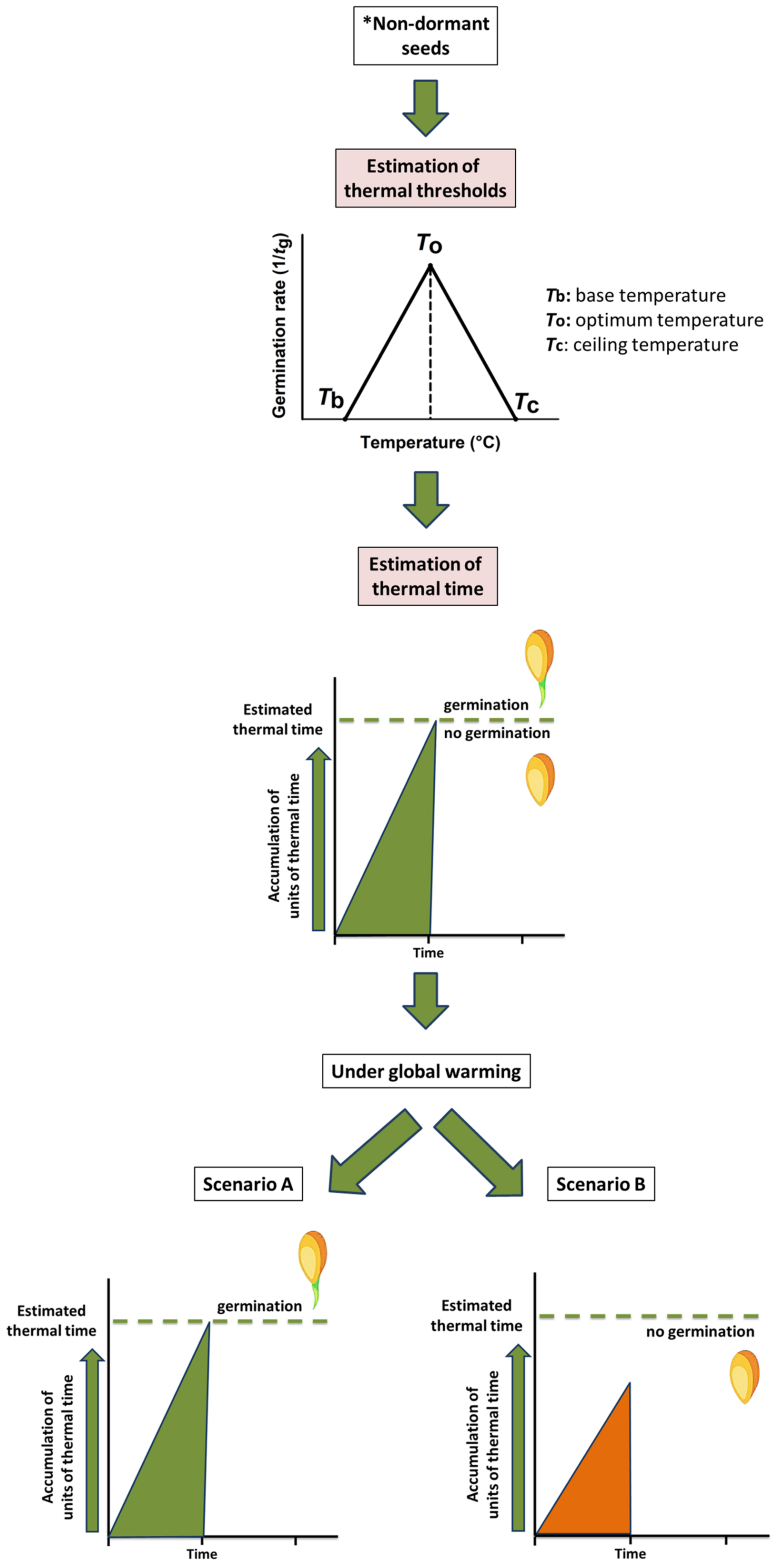
Modelling of thermal time approach to study the germination process of non-dormant seeds. The conceptual figure represents the modelling of thermal time (θ) approach to study the germination process of non-dormant seeds*. Cardinal temperatures representatives of this process are base (*T*_b_), optimal (*T*_o_) and ceiling (*T*_c_) temperatures. Estimation of these values contributes to detecting the number of thermal time units need to be accumulated for germination to occur. Under global warming, seeds could reach the thermal time allowing germination (Scenario A) or could be limited due to the reduction of the accumulated thermal time (Scenario B). *Including seeds in which dormancy was previously removed.

Germination response of non-dormant seeds is modelled by a thermal time approach (García-Huidobro, Monteith & Squire, 1982). This approach (see Fig. 1), for example, has been used to characterise the seed germination niche for detecting plants at higher risk from global warming (Cochrane, Daws & Hay, 2011; Porceddu et al., 2013) and to evaluate the seed germination phenology under the predicted climate change scenarios (Orrù et al., 2012; Porceddu et al., 2013). To understand the thermal time requirements of a species, it is necessary to detect the thermal thresholds (Fernández-Pascual, Mattana & Pritchard, 2019) such as the minimum temperature (*T*_b_, base temperature) and the thermal time (θ) for seed germination (see Fig. 1). In detail, in the thermal time model, seeds accumulate thermal time units, expressing them using a thermal time scale (degree-days, ‘°Cd’; García-Huidobro, Monteith & Squire, 1982; Hardegree, 2006; Orrù et al., 2012; Porceddu et al., 2013, 2017; Seal et al., 2017), to germinate for a percentile (*g*) of the population (García-Huidobro, Monteith & Squire, 1982). To better define the concept (see Fig. 1), the germination process presents an optimal temperature (*T*_o_) at which its rate is maximum; then it progressively decreases below and above this optimum until the temperature reaches the *T*_b_ and the ceiling temperature (*T*_c_), beyond which the germination process ends (García-Huidobro, Monteith & Squire, 1982). The cardinal temperatures representative of this process are *T*_o_, *T*_b_ and *T*_c_, and a certain number of thermal units (θ, °Cd) must accumulate, within the limits of the cardinal temperatures for the process to be completed (Fernández-Pascual, Mattana & Pritchard, 2019 and references therein). These threshold models have been employed successfully to predict field emergence of wild species (Orrù et al., 2012; Porceddu et al., 2013; Ordoñez-Salanueva et al., 2015; Mattana et al., 2017) and also for agricultural purposes (Finch-Savage, 2004).

Many *Gentiana* species have a poorly developed embryo, and some of them need to experience a prolonged period of cold stratification in order to germinate (Nikolaeva, Gladkova & Razumova, 1985; Kohlein, 1991; Cuena-Lombraña et al., 2018a); consequently, the effects of increased temperatures during the cold period or the reduction of the snow cover period on seed dormancy breaking could compromise their seed germination capacity and seedling establishment (Cuena-Lombraña et al., 2018a). In this study, we focused our attention on *Gentiana lutea* L. subsp. *lutea*, whose seeds need temperatures of about 0 °C to break their morphophysiological dormancy (Cuena-Lombraña et al., 2018a). The seed germination requirements of this taxon were deeply studied, both in field (Cuena-Lombraña et al., 2016) and under controlled condition (Cuena-Lombraña et al., 2016, 2018a). However, we decided to carry out this study in order to understand the seed germination behaviour under a cold stratification reduction, and also to supplement the limited knowledge about the thermal thresholds for seed germination for wild native species present in the literature. Indeed, as well as being a species with a high conservation importance, *G. lutea* deserves special attention because it is listed in Annex V of the Habitats Directive (92/43/EEC).

Considering this background, the aims of this work were to (1) evaluate the effect of different cold stratification periods on seed dormancy release and seed germination of *G. lutea* subsp. *lutea*, (2) individuate the cardinal temperatures (i.e. base temperature, optimal temperature and ceiling temperature) and the thermal requirements for seed germination of this taxon using a thermal time approach and (3) estimate the consequences of the temperature rise for its future seed germination behaviour through the RCP projections of climate change scenarios indicated by the IPCC. On these bases, our hypothesis is that a reduction of cold stratification can affect the germination capacity of *G. lutea* subsp. *lutea* through the compromising of seed dormancy breaking. Additionally, we assume that, in the next decades, characterised by considerably warmer temperatures, the germinability of *G. lutea* subsp. *lutea* seeds might be even more limited due to the reduction of the accumulated thermal time.

## Materials and Methods

### Study area

*Gentiana lutea* subsp. *lutea* (hereafter *G. lutea*) is mainly present in the Central-Southern European mountain ranges (Renobales, 2012). In Sardinia (Italy), it grows on the edge of its distributional and ecological range (Fois et al., 2015). Its distribution and its individuals are considered as a panmictic population based on recent genetic studies (Dettori et al., 2018). In detail, the distribution of *G. lutea* in Sardinia consists of only one population and it is restricted to a small area above 1,200 m a.s.l. in the Gennargentu massif (Fois et al., 2015). The annual mean temperature of the Gennargentu Massif varies from ca. 12 °C (at ca. 1,000 m a.s.l.) to ca. 7 °C in the higher areas (at ca. 1,800 m a.s.l.), with a snowfall period lasting from 3 to 4 months (Secci et al., 2010; Cuena-Lombraña et al., 2016, 2018a). The two collection sites, named Is Terre Molentes (IS) and Trainu Murcunieddu (TM), constitute the representative Sardinia natural growing sites of this species. Both are found at an altitudinal range of 1,300–1,500 m a.s.l. Is Terre Molentes is exposed to the northeast and Trainu Murcunieddu to the northwest (Cuena-Lombraña et al., 2016). The sites are characterised by siliceous metamorphic substrate and by an open grassland vegetation type (Cuena-Lombraña et al., 2016).

### Study species and seed lot details

*Gentiana lutea* is an herbaceous rhizomatous perennial plant. The rhizome develops an approximately 80-cm-high flowering stalk during late spring/early summer, with 4–8 oblong leaves, forming a basal rosette (Renobales, 2012). The fruits consist of many-seeded capsules that ripen during the summer, while seed dissemination takes place in August by anemochory (Cuena-Lombraña et al., 2018a). After dispersal, the seeds are exposed first to a slightly warm post-dispersal period before winter begins, and then to low soil winter temperatures (near 0 °C for ca. three months, due to the conditions under the snow cover), while germination occurs in the following spring (Cuena-Lombraña et al., 2016). *G. lutea* seeds have linear underdeveloped embryos and show a morphophysiological dormancy (MPD) with intermediate complex level (Cuena-Lombraña et al., 2018a).

For these experiments, ca. 30 mature fruits (capsules) per individuals of *G. lutea* were collected from at least 50 randomly selected plants in each site, during August 2016 (Authorisation reference: DPM/5D/2005/26104; Ministry of the Environment, Land and Sea; Italy). They were manually cleaned, and well-developed seeds were selected in the laboratory, discarding any visually malformed seeds, and maintained at room temperature (ca. 40% of relative humidity and 20 °C) until the start of the experiments. Seeds were sampled at the same time in two representative localities: one located at 1,460–1,575 m a.s.l. (Is Terre Molentes, IS) and the other one located at 1,324–1,372 m a.s.l. (Trainu Murcunieddu, TM).

### Seed germination tests

There were five cold stratification pre-treatments at 1 ± 1 °C applied to *G. lutea* fresh seeds (600 seeds per treatment per site), following an experimental design that took into account the possible effect of the reduction of the cold period during winter on dormancy release. Concretely, the following pre-treatments were applied: (I) cold stratification for 0 days (C0), (II) cold stratification for 15 days (C15), (III) cold stratification for 30 days (C30), (IV) cold stratification for 60 days (C60) and (V) cold stratification for 90 days (C90). In order to simulate the snow cover period, the chilling pre-treatments were performed in dark conditions (0 h light/24 h dark) (Cuena-Lombraña et al., 2018a). Pre-treatments started at the same time within two weeks after harvesting. Four replicates of 25 randomly selected seeds (maintaining separate the seeds from the different collection sites) were sown in 90-mm diameter plastic Petri dishes for each experimental condition with 1% agar water substrate and incubated with a day/night cycle (12 h light/12 h dark) at 5, 10, 15, 20, 25 and 30 °C in germination chambers (Sanyo MLR-351) with white fluorescent lamps (FL40SS.W/37 70–10 μmol m^−2^ s^−1^). Following the methodology used for *G. lutea* in Cuena-Lombraña et al. (2018a), the germination was checked three times a week, and germinated seeds were scored when a radicle ≥1 mm long was visible. After a minimum period of 90 days, if no additional germination had occurred for two further weeks, non-germinated seeds were cut with a scalpel in order to determine the number of filled, viable and empty seeds (Bacchetta et al., 2006; ISTA, 2008). For germination details, see the Supplemental File.

### Data analysis

For each germination trial, the final germination percentage was calculated on the basis of the filled seeds, excluding the empty seeds, and reported as the mean of the four replicates (±SD). All non-dormant seeds that germinated after different cold stratification periods were considered for the thermal time analyses.

On the basis of the germination rate responses obtained from 10% to 90% of germination, the time estimates (*t*_g_, days) required to reach different cumulative germination percentiles (*g*; with increments of 10% germination) were interpolated from the germination progress curves (Covell et al., 1986). Using a linear model, the germination rate (1/*t*_g_) was regressed according to equation ‘1/*t*_g_ (days^−1^) = (*T*_g_ – *T*_b_)/θ’ as a function of temperature (García-Huidobro, Monteith & Squire, 1982). In order to establish the *T*_b_ for each pre-treatment, a mean (±SD) of the *x*-intercept among percentiles was calculated under the suboptimal temperature range, for example when the germination rate increases linearly with temperature to an optimum temperature (*T*_o_) and, above which, the germination rate starts to decrease (Ellis et al., 1986; Pritchard & Manger, 1990). Due the very low germination percentages obtained after C0, C15 and C30, it was possible to fit the linear regression model only for C60 and C90. Accordingly, the base temperature was possible to estimate only for seeds that germinated after the pre-treatments C60 and C90. The *T*_b_ of each locality and pre-treatment, estimated as the mean value of the *x*-intercepts of the percentiles for which regression lines presented a *P* < 0.05, were considered. The simple linear regression for the germination rate for each percentile, constrained to pass through *T*_b_, were recalculated (Hardegree, 2006). The best-adjusted model resulting in the smallest residual variance was considered (Covell et al., 1986). Thermal times (θ, °Cd) were calculated as the inverse of the suboptimal regression equations (Covell et al., 1986).

### Statistical analyses

Generalised linear models (GLMs) with a quasi-binomial distribution and logit link function were used to evaluate the effect of pre-treatments, temperatures and localities (independent variables) on germination percentages (dependent variable), while a GLM with a quasi-Poisson distribution and logarithmic-link function (log-link) was used for analysing the base temperature (*T*_b_). On the subsequent ANOVA, *F* tests with an empirical scale parameter instead of chi-squared was used (Crawley, 2007). Then significant differences among treatments, temperatures and *T*_b_ values were analysed by a post-hoc pairwise comparisons *t*-test (with Bonferroni adjustment). Statistical analyses were conducted using R version 2.14.1 (R Development Core Team, 2015).

### Projections for future seed germination under climate change conditions

**Figure 2:**
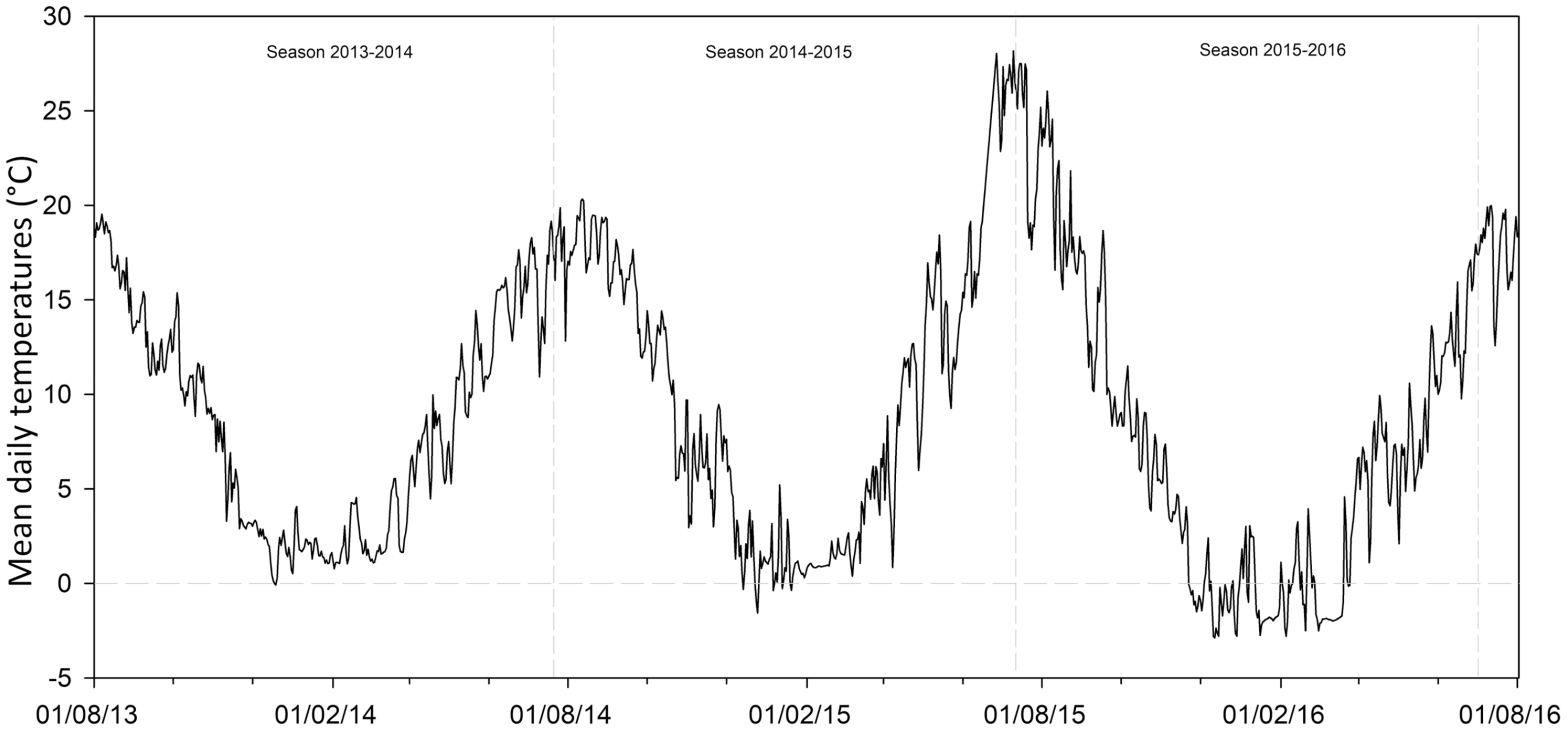
Soil temperatures recorded by data loggers. Mean daily temperatures in both the studied localities from 01/08/2013 to 01/08/2016. Data obtained by data loggers buried in the soil at a depth of ca. 3 cm.

This study also evaluated the potential changes for the future seed germination of *G. lutea* across the different scenarios proposed by the IPCC Team, Pachauri & Meyer (2014). In detail, we considered the four RPC emission scenarios (i.e. RCP2.6, RCP4.5, RCP6.0 and RCP8.5), which represent the different estimated greenhouse gas emissions (IPCC Team, Pachauri & Meyer, 2014). For each of them we considered the low and the high global mean surface temperature predicted, understood as optimistic projections (OP) and less optimistic projections (LOP), respectively. Concretely, we used (I) for RCP2.6, the temperature +0.3 °C and +1.7 °C; (II) for RCP4.5, +1.1 °C and +2.6 °C; (III) for RCP6.0, +1.4 °C and +3.1 °C and (IV) for RCP8.5, +2.6 °C and +4.8 °C. In order to obtain these data and to try to estimate the effect of temperature rise on seed dormancy release and germination of *G. lutea*, we analysed data recorded by soil data loggers (TidbiTw v2 Temp logger; Onset Computer Corporation, Cape Cod, MA, USA) sowed in both the natural localities from 2013 to 2016 (Fig. 2). Using the onset HOBOware PRO (software for HOBO data loggers & devices, Version 3.7.8, Onset Computer Corporation 2002–2016), we extracted the number of days with temperatures ≤1 ± 1 °C recorded for each locality (i.e. the temperature detected for dormancy release; Cuena-Lombraña et al., 2018a). Afterwards, we increased the temperature following the RCPs as detailed above and estimated the number of days with temperatures ≤1 ± 1 °C for each of them in the two sites under study, in order to understand if the temperature required for dormancy release persisted under the predicted increase temperature conditions.

## Results

### Germination behaviour

**Figure 3:**
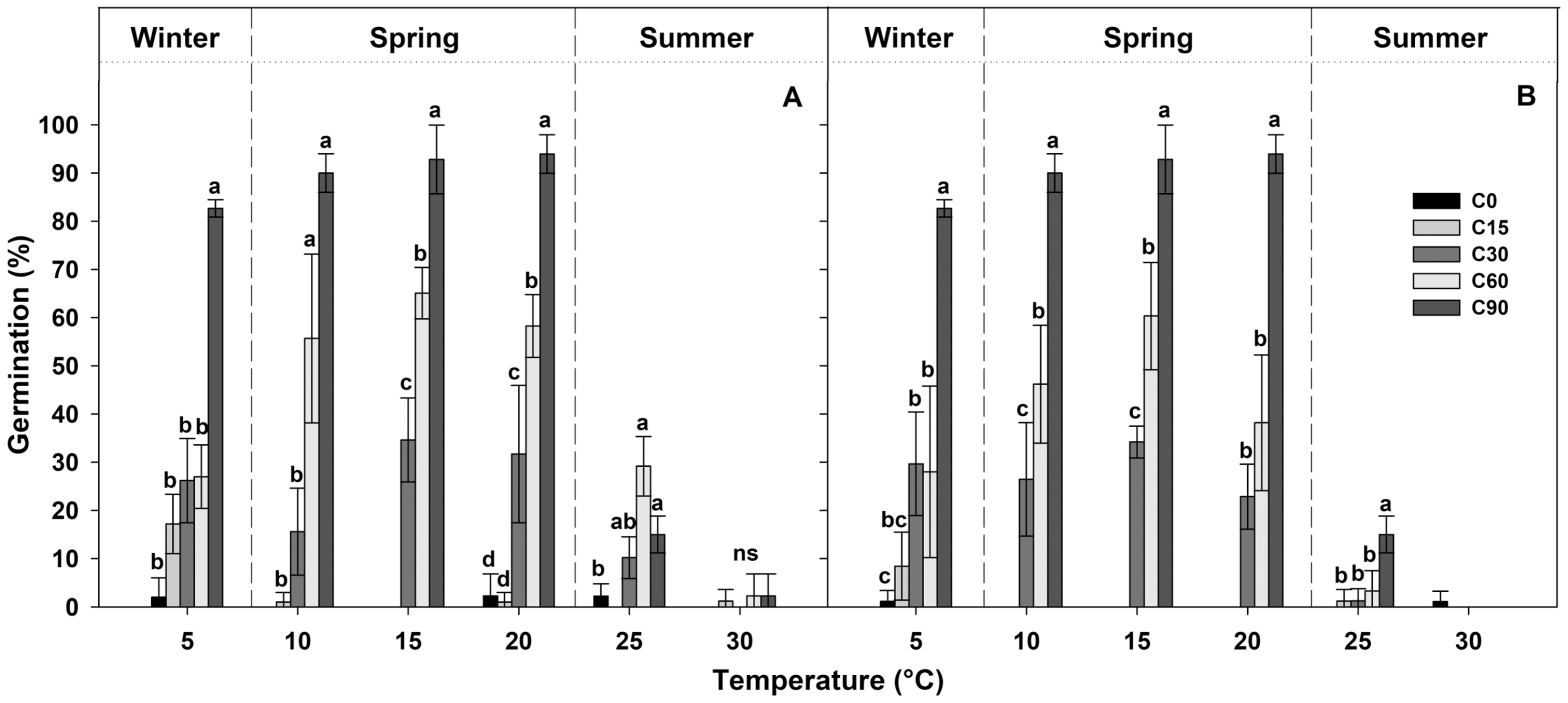
Germination percentages at the end of each treatment. Final germination percentages achieved at the end of the germination tests after each different period of cold stratification (from 0 to 90 days of pre-treatment (C0, C15, C30, C60 and C90)) for (A) IS ‘Is Terre Molentes’ and (B) TM ‘Trainu Murcunieddu’. Data are the mean of four replicates (±SD). Post hoc pairwise *t* test comparisons in each locality were carried out for each germination temperature, and bars with different letters indicate significant (*P* < 0.05) differences among pre-treatments.

Table 1 shows the final germination percentage obtained at the end of each germination test at each temperature tested for seeds collected from the two localities. Seeds pre-treated for 90 days at 1 ± 1 °C (C90) achieved high germination percentages (more than 80%), while, under the other pre-treatments, the germination percentages decreased proportionally with the duration of the pre-treatment (Fig. 3). In the case of C60, the germination percentages were lower than 60%, in the C30 pre-treatment, they were lower than 40%, and, in C15 and C0 pre-treatments, they were lower than 20% (Fig. 3).

**Table 1:** Seed germination results, *T*_b_ and thermal time (θ) after cold stratification pre-treatments. Germination percentages, *T_b_* and thermal time (θ), achieved at the end of the germination tests after each different period of cold stratification (from 0 to 90 days of pre-treatment (C0, C15, C30, C60 and C90)) at each temperature tested for the two localities. *T*_b_ and θ were possible to estimate only for seeds germinated after C60 and C90.

Locality	Temperature (°C)	C0			C15			C30			C60			C90		
		Germination (% ± SD)	*T*_b_ (°C)	θ (°Cd)	Germination (% ± SD)	*T*_b_ (°C)	θ (°Cd)	Germination (% ± SD)	*T*_b_ (°C)	θ (°Cd)	Germination (% ± SD)	*T*_b_ (°C)	θ (°Cd)	Germination (% ± SD)	*T*_b_ (°C)	θ (°Cd)
IS (Is Terre Molentes)	5	2.00 ± 4.00	−	−	17.21 ± 6.16	−	−	26.20 ± 8.75	−	−	27.01 ± 6.60	3.34 ± 1.03	115	82.67 ± 1.81	−1.96 ± 1.23	130
	10	0.00 ± 0.00			1.00 ± 2.00			15.62 ± 9.01			55.70 ± 17.52			90.00 ± 4.00		
	15	0.00 ± 0.00			0.00 ± 0.00			34.63 ± 8.74			65.08 ± 5.32			92.83 ± 7.13		
	20	2.27 ± 4.55			1.00 ± 2.00			31.71 ± 14.24			58.25 ± 6.52			93.96 ±3.97		
	25	2.22 ± 2.57			0.00 ± 0.00			10.20 ± 4.34			29.17 ± 6.18			15.00 ± 3.83		
	30	0.00 ± 0.00			1.19 ± 2.38			0.00 ± 0.00			2.27 ± 4.55			2.27 ± 4.55		
TM (Trainu Murcunieddu)	5	1.14 ± 2.27	−	−	8.46 ± 7.06	−	−	29.67 ± 10.75	−	−	28.01 ± 17.79	2.15 ± 0.86	112	82.67 ± 1.81	−1.58 ± 3.74	108
	10	0.00 ± 0.00			0.00 ± 0.00			26.47 ± 11.77			46.20 ± 12.25			90.00 ± 4.00		
	15	0.00 ± 0.00			0.00 ± 0.00			34.21 ± 3.29			60.33 ± 11.13			92.83 ± 7.13		
	20	0.00 ± 0.00			0.00 ± 0.00			22.86 ± 6.75			38.20 ± 14.10			93.96 ± 3.97		
	25	0.00 ± 0.00			1.19 ± 2.38			1.25 ± 2.50			3.31 ± 4.18			15.00 ± 3.83		
	30	1.09 ± 2.17			0.00 ± 0.00			0.00 ± 0.00			0.00 ± 0.00			0.00 ± 0.00		

In general, the germination of *G. lutea* seeds decreased remarkably at high temperatures (25 and 30 °C, Fig. 3), and, regardless of the pre-treatments, these conditions differed statistically from the temperature range comprised among 5 and 20 °C (Fig. 3).

GLM results indicated that pre-treatments and temperatures factors and their interaction had a statistically significant effects on germination (Table 2), highlighting that there was an interaction between the explanatory variables. The results allowed to highlight that the seeds of *G. lutea* responded differently to the pre-treatments and temperatures tested. On the specific analysis carried out on the pre-treatments (C0, C15, C30, C60 and C90), GLM highlighted that significant differences were among them (Table 2). The post-hoc analysis indicated that there were no statistical differences in the germination response between the treatments C0 and C15 (Table 3), while the differences among C0 and C15 and the longer pre-treatments were significant in all cases (Table 3). In the other cold stratification pre-treatments, statistically significant differences were highlighted among C30, C60 and C90 pre-treatments (Table 3).

**Table 2:** Generalised linear model results of seed germination. GLM results of final seed germination for the following factors: Pre-Treatment (C0, C15, C30, C60 and C90), Temperature (5, 10, 15, 20, 25 and 30 °C) and localities (IS ‘Is Terre Molentes’ and TM ‘Trainu Murcunieddu’) and their interactions.

Germination (%)	Df	Deviance	Resid. Df	Resid. Dev	*F*	*P* (>*F*)
NULL			239	13,627.1		
Pre-treatment	4	7,194.8	235	6,432.3	432.8539	<2.2^e−16^***
Temperature	5	4,415.5	230	2,016.7	212.5184	<2.2^e−16^***
Locality	1	5.5	229	2,011.2	1.3230	0.2516
Pre-treatment × temperature	20	832.7	209	1,178.5	10.0198	<2.2^e−16^***
Pre-treatment × locality	4	121.3	205	1,057.2	7.2972	1.812^e−05^***
Locality × temperature	5	185.9	200	871.3	8.9467	1.312^e−07^***
Pre-treatment × locality × temperature	20	103.4	180	767.9	1.2447	0.2229

**Note:**

Significance of *P* values codes: ‘***’<0.05.

**Table 3:** Pairwise comparisons on different cold stratification period. Pairwise comparisons using *t* tests with pooled statistical differences for final germination results data under different cold stratification period (C0, C15, C30, C60 and C90 correspond to 0, 15, 30, 60 and 90 days of cold stratification at 1 ± 1 °C).

	C0	C15	C30	C60	C90
C0	–	1.00000	0.00036***	5.9^e−12^***	<2^e−16^***
C15	1.00000	–	0.00177***	7.0^e−11^***	<2^e−16^***
C30	0.00036***	0.00177***	–	0.00754***	2.5^e−16^***
C60	5.9^e−12^***	7.0^e−11^***	0.00754***	–	2.7^e−07^***
C90	<2^e−16^***	<2^e−16^***	2.5^e−16^***	2.7^e−07^***	–

**Note:**

Significance of *P* values codes: ‘***’<0.05.

### Base temperature for seed germination and thermal time requirements

**Figure 4:**
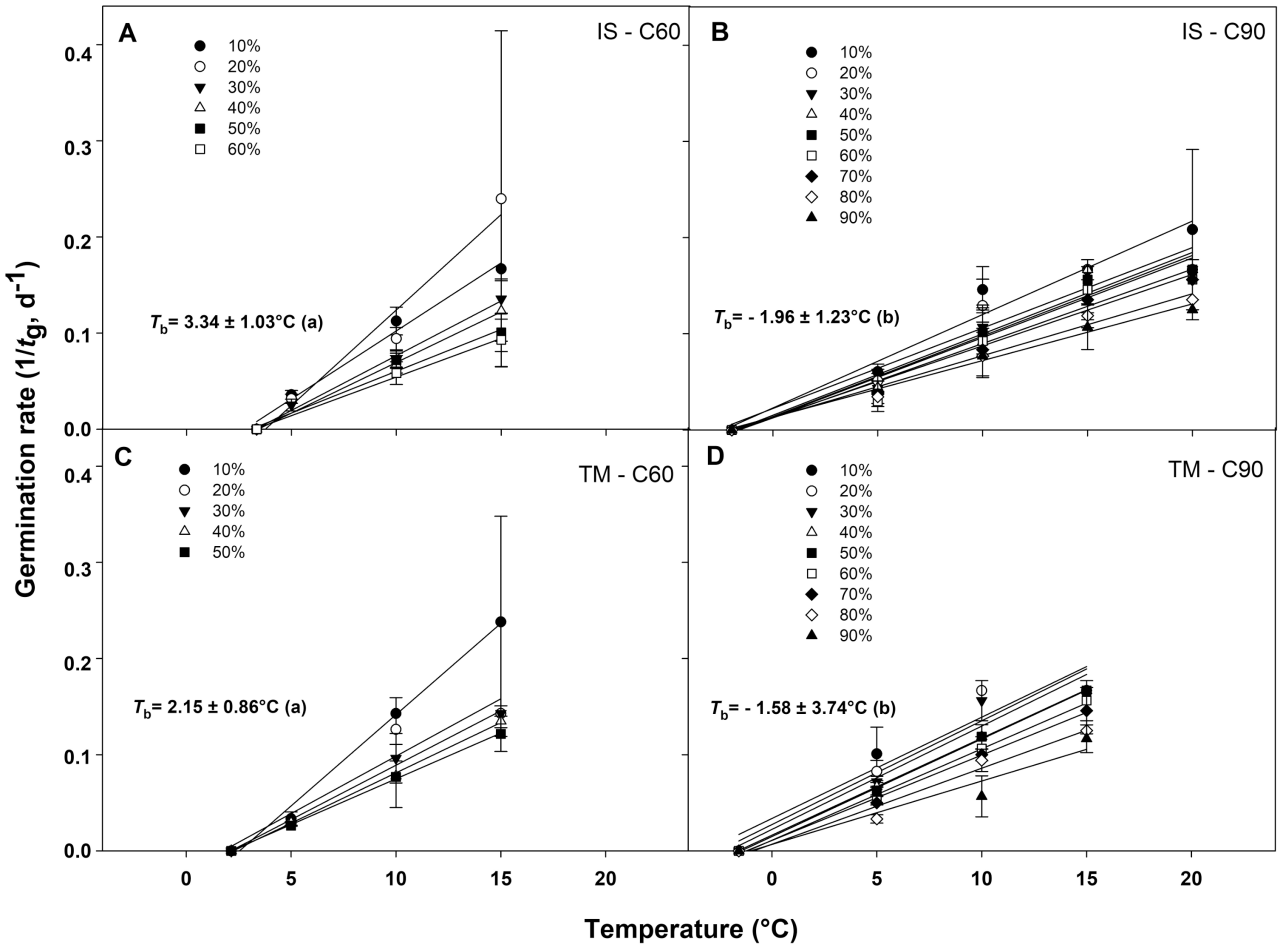
Base temperatures for seeds germination of *G. lutea*. Base temperatures (*T*_b_) for *G. lutea* seeds germination of the two studied localities ((A and B): IS ‘Is Terre Molentes’ and (C and D): TM ‘Trainu Murcunieddu’), calculated after the C60 (60 days at 1 ± 1 °C; A and C) and C90 (90 days at 1 ± 1 °C; B and D) pre-treatments. Within each locality and pre-treatment, linear regressions for the different percentiles were constrained to the common value of *T*_b_. *T*_b_ values were not calculated for percentiles whose regression lines had *P* > 0.05. *T*_b_ values with different letters are significantly different at *P* < 0.05.

**Figure 5:**
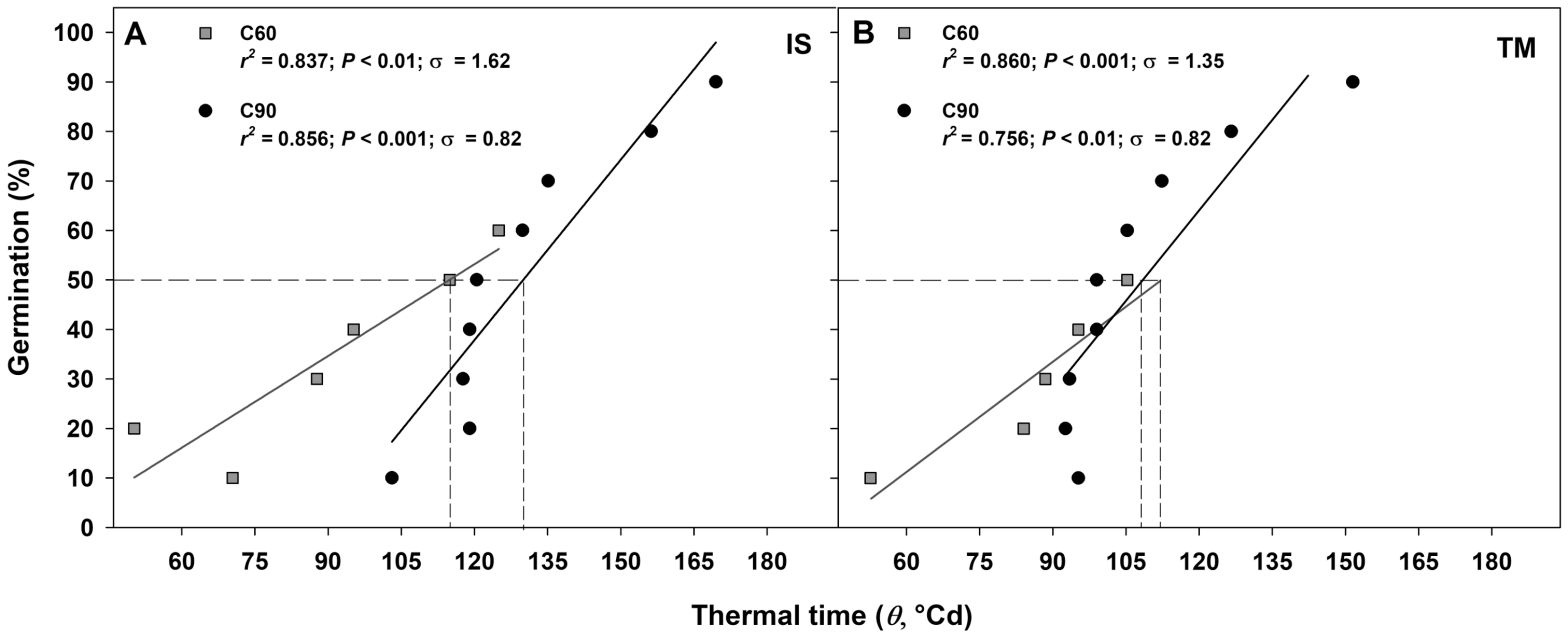
Thermal time requirement after 60 and 90 days at 1 ± 1 °C. Germination after C60 (60 days at 1 ± 1 °C) and C90 (90 days at 1 ± 1 °C) for the two studied localities (A: IS ‘Is Terre Molentes’ and B: TM ‘Trainu Murcunieddu’) as a function of the thermal time requirement (θ, °Cd). Thermal times were calculated from germination time courses, assuming the common *T*_b_ reported in Fig. 3. Thermal times that required reaching 50% of germination (θ_50_) are shown with dashed lines.

The germination rate responses obtained from 10 to 50−60% and from 10% to 90% permitted to fit the linear regressions of the relationship between the temperatures and the rate of germination (1/*t*_g_) for seed germinated after C60 and C90, respectively (Figs. 4A–4D). In accordance to the goodness of fit (*r*^2^), the sub-optimal temperature for TM encompassed only data up to 15 °C (Figs. 4A and 4C), while for IS, the temperature 15 °C (in C60) and 20 °C (in C90) were included in the sub-optimal temperature (Figs. 4B and 4D). The *T*_b_ for C60 were 3.34 ± 1.03 °C for IS and 2.15 ± 0.86 °C for TM (Figs. 4A and 4C), while the *T*_b_ for C90 were −1.96 ± 1.23 °C and −1.58 ± 3.74 °C for IS and TM, respectively (Figs. 4B and 4D). The mean *T*_b_ estimated values showed no statistical differences between localities (Fig. 4). However, significant differences were detected among pre-treatments (Fig. 4). In both localities, an increase of *T*_b_ with the decrement in the duration of the cold stratification was detected (Fig. 4). In IS, the thermal time required for 50% of germination (θ_50_) was slightly greater in C90 than C60 (Fig. 5). In TM, θ_50_ was slightly greater in C60 compared to C90 (Fig. 5). However, in general, θ_50_ were similar for both localities and, after both cold stratification periods, with values that ranged from ca. 108 °Cd to ca. 130 °Cd (Fig. 5; Table 1).

### Projections for the future seed germination under climate change conditions

Using the RCP scenarios and extrapolating the soil data information from the studied localities (Fig. 2), the total number of days with temperature ≤1 ± 1 °C were calculated (see Table 4). Starting from the current data and analysing the temperature recorder by data logger from the year 2013 to 2016, the number of days with temperature ≤1 ± 1 °C was 76.33 ± 14.36 in IS and 78.33 ± 12.66 in TM (see Table 4). Incrementing the temperature following the RCP scenarios, it was possible to detect that under the predicted temperature change scenarios, the number of days will decrease in both localities. In detail, under the RCP2.6 scenario, seeds of *G. lutea* could break their dormancy (ensuring the germination) only in the case of the increment of +0.3 °C (optimistic projection), in which there will be present at least 60 days of temperature ≤1 ± 1 °C (Table 4). In the same scenario but with the increment of +1.7 °C (less optimistic), the number of days with temperature ≤1 ± 1 °C will decrease to less than 30 days (Table 4), which will lead to a condition that will not permit the completion of dormancy release, compromising the germination capacity of *G. lutea* seeds. Under the RCP 4.5 scenario, the increment of +1.1 °C predicted for the optimistic projection, the number of days with temperature ≤1 ± 1 °C will be ca. 35–40 days (see Table 4), which could permit too few seeds to germinate (ca. 30%; see Fig. 3). Under the others RCP scenarios (i.e. RCP6.0 and RCP8.5), both in the optimistic and less optimistic ones, the number of days with temperature ≤1 ± 1 °C will be very low (Table 4).

**Table 4:** Days with temperature ≤1 ± 1 °C. Number of days with temperature ≤1 ± 1 °C under the predicted temperature change scenarios (sensu IPCC Team, Pachauri & Meyer, 2014) for Is Terre Molentes (IS) and Trainu Murcunieddu (TM) localities from 2013 to 2016.

Locality	IPCC scenario	Increase in temperature (°C)	Mean number of days with temperature ≤1 ± 1 (°C)
IS	Current	–	76.33 ± 14.36
	RCP 2.6	+0.3 (OP)	68.33 ± 24.00
		+1.7 (LOP)	18.00 ± 14.56
	RCP 4.5	+1.1 (OP)	33.66 ± 23.86
		+2.6 (LOP)	4.33 ± 4.51
	RCP 6.0	+1.4 (OP)	24.00 ± 15.72
		+3.1 (LOP)	0.66 ± 1.15
	RCP8.5	+2.6 (OP)	4.33 ± 4.51
		+4.8 (LOP)	0
TM	Current	–	78.33 ± 12.66
	RCP 2.6	+0.3 (OP)	73.33 ± 15.31
		+1.7 (LOP)	20.00 ± 11.00
	RCP 4.5	+1.1 (OP)	39.00 ± 15.52
		+2.6 (LOP)	7.66 ± 10.02
	RCP 6.0	+1.4 (OP)	28.00 ± 8.71
		+3.1 (LOP)	1.00 ± 1.73
	RCP 8.5	+2.6 (OP)	7.66 ± 10.02
		+4.8 (LOP)	0

**Note:**

OP, optimistic projections; LOP, less optimistic projections.

## Discussion

### Effect of reduction of cold stratification period on seed germination

The responses to different durations of the cold stratification period indicated that *G. lutea* needs at least 60 days of cold pre-treatment in order to release seed dormancy and reach a successful germination (understood as more than 50% of germination). In detail, our study detected some critical issues related to the number of days with cold period or snow cover that allow us to hypothesise that the capacity of germination of *G. lutea* seeds will be drastically reduced in the future as a consequence of the global warming trend. Although it is true that the reproductive phase offers the plant species the opportunity to adapt gradually to environmental changes (Hedhly, Hormaza & Herrero, 2009), the changes that plants are currently experiencing are occurring too fast to have enough time to adapt to the temperature change (Hedhly, Hormaza & Herrero, 2009). In this context, the results obtained here for *G. lutea* appeared in contrast with that obtained for *Rhamnus persicifolia* Moris seeds, in which a reduction of the cold stratification period from 90 days to 30 days would not be detrimental per se for seed germination, although under 30 days of cold stratification, the seed germination was importantly reduced (Porceddu et al., 2013). This aspect permits the highlighting that different plants growing in similar climatic conditions may respond differently to the rapid climate changes and that some species may suffer the effects of climate change before others.

In the face of the current climate change, the migration and persistence of plants depends on their successful reproduction by seeds, a fundamental aspect of the life cycle of the plant that affects population performance, as well as plant species distribution and community assembly (Fernández-Pascual, Mattana & Pritchard, 2019). Consequently, *G. lutea* and other species that need minimum days of cold temperatures to promote their seed dormancy release and germination could be more threatened in the future, unlike species without such requirements. Species growing in the same Sardinian areas of *G. lutea* may have similar thermal requirements for seed dormancy release and germination, responding similarly to global warming. On the other hand, however, other species that occupy the same areas may require different conditions, like higher temperatures for dormancy breaking, causing consequently different germination behaviour with respect to the seeds of *G. lutea*. For example, Sardinian species growing at the same altitudinal range (e.g. from 1,200 to 1,350 m a.s.l.) may reduce the capacity of dormancy release under one month at a temperature of 5 °C (e.g. in *R. persicifolia*; Porceddu et al., 2013), while a warm condition as temperature of 25 °C for three months may promote the dormancy release allowing the seed germination (e.g. in *Ribes sandalioticum* (Arrigoni) Arrigoni and *Ribes sardoum* Martelli, Mattana et al., 2012; Porceddu, Fenu & Bacchetta, 2017). As reported by Briceño, Hoyle & Nicotra (2015) and Walder & Erschbamer (2015) some species could be indirectly favoured by a future warming climate, resulting in significant changes in the composition and dynamics of different plant communities. All this suggests that a deeper study on a wide scale may help in understanding if other species distributed with *G. lutea* will be affected by global warming in a similar way.

Theoretically, regeneration processes are more suitable in the centre of the species’ distribution range than in the natural distribution limits (Lawton, 1993). For this reason, viability and regeneration evaluations of peripheral populations of plants under different climate change scenarios have become a conservation concern (Giménez-Benavides, Escudero & Iriondo, 2007). In this study, focused on the populations of *G. lutea* located at the edge of its distributional and ecological range, the germination responses highlighted a critical decrease of the germination percentage with the reduction of a cold stratification period. This could indicate that these populations, which are located at lower altitudes and at the edge of its distributional and ecological range, would be more threatened, if facing shorter winters. Seed germination results obtained in this work, in particular, the detection that seeds of *G. lutea* needed at the least 60 days of cold temperature to remove dormancy, are in agreement with previous studies (Fois et al., 2016; Cuena-Lombraña et al., 2018b) which reported that the most affected localities are those that are present at the lowest altitudes. Therefore, with the results presented here, it is possible to affirm that, while also considering the germination capacity of the seeds, climate warming will likely first affect the localities of *G. lutea* at lower altitudes.

### Cardinal temperatures and thermal time

The optimal temperatures of non-dormant seeds of *G. lutea* detected in this work is assumed to be around 10−20 °C, as the best models in the sub-optimal range were obtained by considering a temperature from 5 to 20 °C. On the other hand, the temperatures >20 °C were included in the range of supra-optimal temperatures. The latter aspect highlights that the higher temperatures negatively affected the germination capacity of non-dormant seeds of *G. lutea*; it was already the case at 25 and 30 °C, that is the higher temperatures used in this work. This assumption is in accordance with the temperatures (from 10 to 20 °C) suggested in the optimal germination protocol by Cuena-Lombraña et al. (2018a) further confirming that the seeds of this species are characterised by spring germination. The germination of seeds of *G. lutea* occurs, in particular, after the snow melting period in which the risk of frost is low, the availability of water is high and the time of solar radiation is longer. The base temperature (*T*_b_) in *G. lutea* seeds, regardless of the locality, varied from ca. 2 to 3 °C (after 60 days of cold pre-treatment) to ca. −2 °C (after 90 days of cold pre-treatment). Pre-treatments for dormancy release (C60 and C90) allowed the reduction of the *T*_b_ in *G. lutea* seeds. The effect of cold stratification in reducing the *T*_b_ has been also observed in seeds of *Aesculus hippocastanum* L. (Pritchard et al., 1999) and *R. persicifolia* (Porceddu et al., 2013). The reduction of the base temperature should increase the rate of accumulation of thermal units, as it happens in species like *Vitis vinifera* L. subsp. *sylvestris* (C.C. Gmel) Hegi (Orrù et al., 2012) and *R. persicifolia* (Porceddu et al., 2013). However, in seeds of *G. lutea*, the duration of cold stratification periods that promote dormancy release and seed germination (i.e. C60 and C90) do not cause a large variation in their thermal time estimates (θ_50_), whose values were similar under the two conditions. In this sense, after a certain period of cold stratification, seeds of *G. lutea* start to accumulate thermal time units that allow them to germinate and with the reduction of duration of the overwintering season (<60 days), dormancy loss could be compromised. In addition, the thermal time (θ_50_) requirements after brief winters could remain too high, thus potentially preventing seed germination.

### Implications for seed germination under global warming

As far as the ecological implications under climate change scenarios are concerned, the future conditions may not meet the future requirements for breaking physiological dormancy in *G. lutea* seeds and will be detrimental to the proportion of seeds which germinate. Similarly, as reported by Orrù et al. (2012) for *V. vinifera* subsp. *sylvestris*, under the two simulated IPCC scenarios that they considered (e.g. an increase in temperature of +1.8 °C and +3.4 °C), the seed germination could be compromised because the future winters temperature may not be cold enough to break physiological dormancy. Contrary to these responses, an increase in soil temperature is predicted to alter the timing of germination of *Polaskia* Backeb, but this will not be detrimental to the proportion of seeds germination (Ordoñez-Salanueva et al., 2015).

As reported in Table 3, under several RCP scenarios (sensu IPPC, 2014), the number of days with a suitable temperature for dormancy release in *G. lutea* decreased drastically, particularly under the less optimistic projections. As already mentioned in this work, after a certain duration of chilling, seeds of *G. lutea* start to accumulate thermal time units that allow them to germinate. If the future increase in temperatures, as predicted by IPCC, reduces the duration of the overwintering period, dormancy loss and, consequently, seed germination of *G. lutea* could be compromised. In addition, if the autumn/winter season shortens, the thermal time (θ_50_) requirements could remain too high, potentially reducing the likelihood of seed germination. This statement is in agreement with what was reported in the ‘Model D1’ by Fernández-Pascual, Mattana & Pritchard (2019). The same authors reported the importance of considering the thermal memory effect of seed dormancy (see ‘Model D2’; Fernández-Pascual, Mattana & Pritchard, 2019). They suggest that, if a shortened winter causes a decrease in the amount of dormancy loss, thermal memory might be enough to reduce the value of θ_50_ to a level that allows germination at an adequate time. However, in the case of *G. lutea*, we theorise that under several RPC emission scenarios (see Table 3), thermal memory (sensu Fernández-Pascual, Mattana & Pritchard, 2019) may not be enough to adjust its germination phenology to global warming.

In particular, for the populations of *G. lutea* located at the edge of its distributional and ecological range, we hypothesised that increases of more than +1.7 °C would significantly reduce the germination of this species in field conditions. In the literature, we find controversial results related to the germination behaviour of alpine or mountain species under climate change conditions. On the one hand, there are studies which showed that warming can either increase (Milbau et al., 2009; Shevtsova et al., 2009; Mondoni et al., 2015; Wang et al., 2018) or decrease (Jump et al., 2008; Hoyle et al., 2015) germination percentage. The response to the temperature increments varies depending on the plant species (Hedhly, Hormaza & Herrero, 2009) and for this reason, it is particularly important to study each species individually. The consequences of a warming environment on plant reproduction cannot be simply interpreted qualitatively as a series of absolute positives and negatives (Fernández-Pascual, Mattana & Pritchard, 2019). The effect of temperature rise should instead be understood in terms of the variation of the rates and thresholds that quantify the physiological processes underlying reproduction by seed (Fernández-Pascual, Mattana & Pritchard, 2019).

We might expect that a period of cold stratification would be an ordinary condition for seed germination to occur in mountain or alpine plant species, but this is not always the case. For example, in the studies carried out by Sommerville, Martyn & Offord (2013) and Hoyle et al. (2015) on 19 and 54 Australian species, respectively, only half of the taxa studied increased their seed germination performance as a result of a cold stratification period. Contrarily, in most of the 27 Japanese alpine species (Shimono & Kudo, 2005) and in alpine plants from the Central Chilean Andes (Cavieres & Sierra-Almeida, 2018), a cold period of stratification was needed to promote seed germination. Moreover, species living in environments with a contrasting snow cover period, as occurs in the Mediterranean mountains, indicate that there is a positive relationship between seed germination response and the length of the snow cover period (Meyer, McArthur & Jorgensen, 1989; Meyer, Kitchen & Carlson, 1995). This aspect is in accordance with what we detected for *G. lutea*, in which the seed germination increased with the increase in length of cold stratification. Infact, our study detected some critical issues related to the number of days with cold temperature, suggesting that the germination of *G. lutea* will be reduced in the future, as a consequence of global warming. In accordance with Briceño, Hoyle & Nicotra (2015) and Walder & Erschbamer (2015), we expected that a further decrease in seedling emergence will occur in the future due to global warming, thus leading to a shift towards asexual reproduction for *G. lutea*. The reproductive phase represents an opportunity for the plant to adapt to a changing environment. Therefore, new knowledge on plant adaptation, plant evolution and vegetation dynamics under a global warming might derive from the understanding of plant plasticity in response to prevailing temperatures and the associated potential changes (Hedhly, Hormaza & Herrero, 2009).

## Conclusions and perspectives

We conclude that seeds of *G. lutea* need at least 60 days of cold stratification to remove dormancy and promote the germination, and that temperatures >20 °C negatively affected the germination of non-dormant seeds. The thermal time model developed in this work allowed us to identify the thermal threshold requirements of seed germination of this species, increasing the knowledges of a plant threatened by global warming. In fact, in incrementing the temperature following the RCP scenarios proposed by IPCC, we estimated that the success of seed germination of *G. lutea* will be seriously compromised. Our results, in addition, emphasise the need for further studies that aim at a better characterisation of germination efficiency, especially for species that require cold stratification. The framework applied in this study might be an important step towards the future development of seed germination models that consider future climatic scenarios and that could be applied not only to Mediterranean mountain species but also to widespread taxa. This would improve the knowledge on the germination mechanisms of adaptation to different future climatic scenarios under global warming conditions, and thus contribute to the development of efficient plant conservation strategies.

## Supplemental Information

10.7717/peerj.8894/supp-1 Supplemental Information 1 *Gentiana lutea* seeds collection form.

10.7717/peerj.8894/supp-2 Supplemental Information 2 *Gentiana lutea* dehydration form.

10.7717/peerj.8894/supp-3 Supplemental Information 3 *Gentiana lutea* cleaning and conservation form.

10.7717/peerj.8894/supp-4 Supplemental Information 4 *Gentiana lutea* image.

10.7717/peerj.8894/supp-5 Supplemental Information 5 Germination percentages obtained at the end of the test. Germination percentages achieved at the end of the germination tests after each different period of cold stratification (C0, C15, C30, C60 and C90).

10.7717/peerj.8894/supp-6 Supplemental Information 6 Soil temperature recorded by data loggers.

10.7717/peerj.8894/supp-7 Supplemental Information 7 Germination rate for base temperature.

10.7717/peerj.8894/supp-8 Supplemental Information 8 Thermal time values.

## Additional Information and Declarations

### Field Study Permissions

The following information was supplied relating to field study approvals (i.e., approving body and any reference numbers): The Ministry of the Environment, Land and Sea, Italy, approved this study (DPM/5D/2005/26104).

### Data Availability

The following information was supplied regarding data availability: Data is available in the Supplemental Files. Data is also available at the BG-SAR and were stored in IrisBG database (data not available on-line): 122/16, 118/16. This information is also available at Porceddu, Marco; Cuena-Lombraña, Alba; Bacchetta, Gianluigi (2020), “*Gentiana lutea* subsp. *lutea* information”, Mendeley Data, v3 DOI 10.17632/mdys8hx3xy.3.
